# New Advertisements

**Published:** 1888-09

**Authors:** 


THE ATTENTION OF OUR READERS is called to the new advertisements of Messrs. Reed & Carnrick, Parke, Davis & Co., McKesson & Robbins, and Dr. G. W. McCray, in this number.

